# Facile synthesis of nano-Li_4 _Ti_5_O_12 _for high-rate Li-ion battery anodes

**DOI:** 10.1186/1556-276X-7-10

**Published:** 2012-01-05

**Authors:** Yun-Ho Jin, Kyung-Mi Min, Hyun-Woo Shim, Seung-Deok Seo, In-Sung Hwang, Kyung-Soo Park, Dong-Wan Kim

**Affiliations:** 1 Department of Materials Science and Engineering, Ajou University, Suwon 443-749, South Korea

## Abstract

One of the most promising anode materials for Li-ion batteries, Li_4_Ti_5_O_12_, has attracted attention because it is a zero-strain Li insertion host having a stable insertion potential. In this study, we suggest two different synthetic processes to prepare Li_4_Ti_5_O_12 _using anatase TiO_2 _nanoprecursors. TiO_2 _powders, which have extraordinarily large surface areas of more than 250 m^2 ^g^-1^, were initially prepared through the urea-forced hydrolysis/precipitation route below 100°C. For the synthesis of Li_4_Ti_5_O_12_, LiOH and Li_2_CO_3 _were added to TiO_2 _solutions prepared in water and ethanol media, respectively. The powders were subsequently dried and calcined at various temperatures. The phase and morphological transitions from TiO_2 _to Li_4_Ti_5_O_12 _were characterized using X-ray powder diffraction and transmission electron microscopy. The electrochemical performance of nanosized Li_4_Ti_5_O_12 _was evaluated in detail by cyclic voltammetry and galvanostatic cycling. Furthermore, the high-rate performance and long-term cycle stability of Li_4_Ti_5_O_12 _anodes for use in Li-ion batteries were discussed.

## Introduction

Li_4_Ti_5_O_12 _is one of the most promising anode materials for Li-ion batteries even though it has lower specific capacity (175 mAh g^-1^) than does graphite (372 mAh g^-1^). One of the unique properties of Li_4_Ti_5_O_12 _is the negligible lattice change in the Li-ion insertion/desertion process, which provides good high-rate cycling stability [1]. The electrochemical properties of Li_4_Ti_5_O_12 _are dependent on its method of preparation. The conventional solid-state, sol-gel [2], hydrothermal [3], spray pyrolysis [4], and combustion [5] methods have been proposed for Li_4_Ti_5_O_12 _synthesis. Among these, the solid-state process is a simple method that is well suited for production scale-up. However, the solid-state process using TiO_2 _as a starting precursor requires lengthy heating with Li salts at high temperatures in order to obtain highly crystalline Li_4_Ti_5_O_12 _[6]. As a result, particle size control is more difficult than that in hydrothermal or sol-gel method, and the resultant larger particles lead to poor capacity retention and rate capability.

Herein, we demonstrate the preparation of highly crystalline nanosized Li_4_Ti_5_O_12 _[nano-Li_4_Ti_5_O_12_] with a uniform particle size via a urea-mediated wet process, in which a TiO_2 _precursor with a large surface area is initially formed, followed by wet and solid-state processes with different Li sources, LiOH and Li_2_CO_3_, respectively. After subsequent heat treatment, the electrochemical performance of the resultant Li_4_Ti_5_O_12 _as an anode for Li-ion batteries is evaluated and discussed.

## Experimental procedure

### Preparation of TiO_2 _precursor

TiO_2 _nanoparticles with an anatase structure were prepared using the urea-mediated precipitation method [7], in which 0.015 M titanium trichloride (20% in 3% hydrochloric acid, TiCl_3_, Alfa Aesar, Ward Hill, MA, USA) and 3.0 M urea (99.3%, (NH_2_)_2_CO, Alfa Aesar, Ward Hill, MA, USA) were dissolved in deionized [DI] water at room temperature. The solution was heated at 90°C to 100°C for 4 h with magnetic stirring. Precipitates were obtained by centrifugation and repeated washing (five times with DI water and once with anhydrous ethanol). The powders were dried at 100°C for several hours in a vacuum oven.

### Preparation of Li_4_Ti_5_O_12_

#### Wet process

Stoichiometric amounts of the prepared TiO_2 _nanopowder were dispersed in DI water by sonication for 2 h. A stoichiometric amount of LiOH (98%, Sigma-Aldrich, St. Louis, MO, USA) was then dissolved in the solution with stirring. The resulting white-colored suspensions were heated at 110°C to evaporate water. Finally, the powder was calcined at various temperatures in air to afford Li_4_Ti_5_O_12_.

#### Solid-state process

For the solid-state process, Li_2_CO_3 _(99%, Sigma-Aldrich, St. Louis, MO, USA) was chosen as the Li source. The stoichiometric mixture was agitated for 24 h with a zirconia ball in absolute ethanol, dried, and calcined at various temperatures in air.

### Characterization of TiO_2 _precursors and Li_4_Ti_5_O_12 _nanoparticles

The powders were characterized by X-ray powder diffraction [XRD] (D/max-2500 V, Rigaku, Tokyo, Japan), Brunauer-Emmett-Teller [BET] (Belsorp-mini II, BEL Japan Inc., Osaka, Japan) surface area determination, high-resolution transmission electron microscopy [HRTEM] (JEM-3000F, JEOL, Tokyo, Japan) at an accelerating voltage of 300 kV, and field-emission scanning electron microscopy [FESEM] (JSM-6700F, JEOL, Tokyo, Japan).

### Electrochemical analysis

A mixture consisting of 70 wt.% of the active materials, 15 wt.% Super P carbon black (MMM Carbon, Brussels, Belgium), and 15 wt.% Kynar 2801 binder (PVDF-HFP, Arkema Inc., King of Prussia, PA, USA) was dissolved in 1-methyl-2-pyrrolidinone (Sigma-Aldrich, St. Louis, MO, USA) solvent for uniform dispersion of the active materials on a Cu foil to obtain positive electrodes. Then, the solvent was evaporated in a vacuum oven at 100°C. A Swagelok-type cell was assembled in an Ar-filled glove box in order to protect the cell from oxidation and moisture. A Li metal foil (negative electrode) and the prepared mixture (positive electrode) were saturated with a liquid electrolyte obtained by dissolving 1 M LiPF_6 _in ethylene carbonate and dimethyl carbonate (1:1 by volume, Techno Semichem Co., Ltd., Sungnam, South Korea). Li_4_Ti_5_O_12 _powders were analyzed by the galvanostatic discharge/charge cycling method and cyclic voltammetry [CV] measurements with a battery cycler (WBCS 3000, WonATech, Seoul, South Korea). Each cell was cycled through a voltage range of 1.0 to 2.5 V versus Li/Li^+^.

## Results and discussion

**Figure 1:**
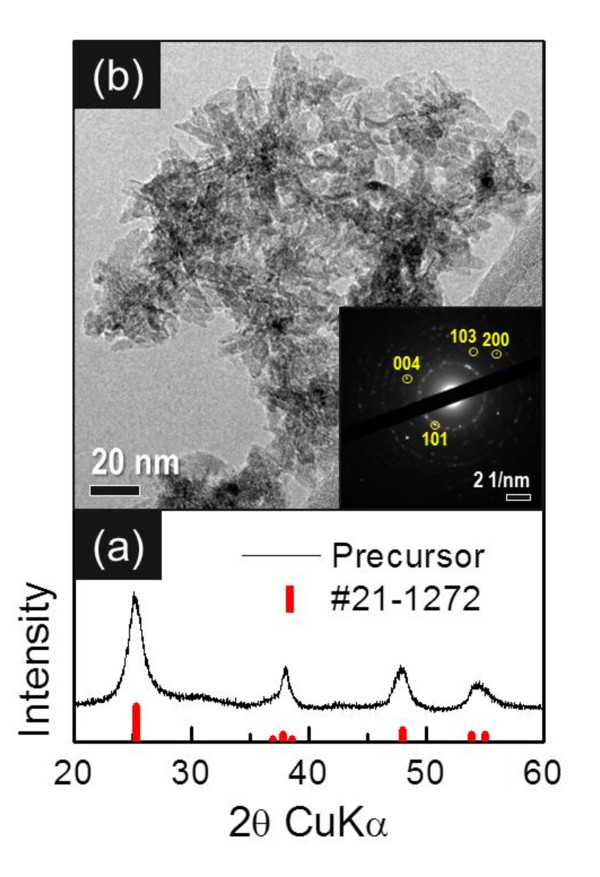
**Characterization of TiO_2 _products**. (**a**) A typical XRD pattern. (**b**) A TEM image of TiO_2 _precursor powders. The inset in (b) shows SAED patterns. (By Jin YH et al.).

The XRD pattern (Figure 1a) for precursor powders indicated that they comprised anatase-phase TiO_2 _(Joint Committee of Powder Diffraction System [JCPDS] #21-1272). The TiO_2 _morphology was found to be flower-like clusters of 50 nm in size, which comprised tiny aggregated nanorods (Figure 1b). For this reason, the powder had an extremely large surface area, 267 m^2 ^g^-1^, as confirmed by BET surface area measurements. In addition, the electron diffraction (selected area electron diffraction [SAED]) pattern of the selected area coincided with that of anatase TiO_2_, as shown in the inset of Figure 1b.

**Figure 2:**
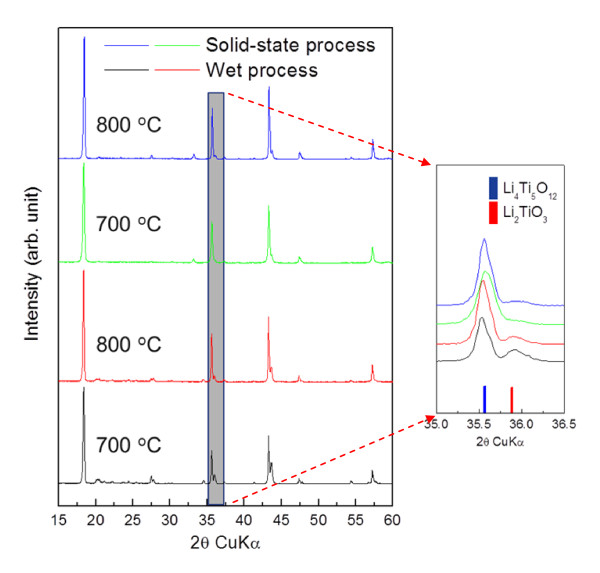
**XRD patterns of Li_4_Ti_5_O_12 _powders**. Li_4_Ti_5_O_12 _prepared through wet and solid-state processes and subsequently calcined at 700°C and 800°C for 4 h. (By Jin YH et al.).

In order to obtain nano-Li_4_Ti_5_O_12 _with a sufficiently large surface area, the TiO_2 _powders prepared as mentioned above were used as precursors. After mixing the TiO_2 _precursor with LiOH and Li_2_CO_3 _through wet and solid-state processes, respectively, both mixtures were calcined at 700°C and 800°C and were found to mainly comprise the cubic Li_4_Ti_5_O_12 _phase (JCPDS #49-0207; Figure 2). However, the Li_4_Ti_5_O_12 _powders prepared through the wet process had an undesirable (Li-inactive) secondary phase, Li_2_TiO_3 _(JCPDS #33-0831), even after calcination at 800°C as confirmed by the XRD peak at 2*θ *= 35.6°. As opposed to the powders prepared by the wet process, those prepared through the solid-state process showed an almost pure Li_4_Ti_5_O_12 _phase with negligible secondary phases.

**Figure 3:**
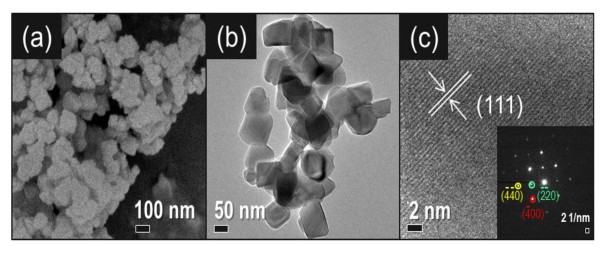
**FESEM and HRTEM images**. (**a**) FESEM image of a typical Li_4_Ti_5_O_12_. (**b**) Low-magnification HRTEM image of Li_4_Ti_5_O_12_. (**c**) HRTEM image of Li_4_Ti_5_O_12 _powders prepared through the solid-state process and subsequently calcined at 700°C for 4 h. The inset in (c) shows SAED patterns. (By Jin YH et al.).

Figures 3a, b show the typical FESEM and HRTEM images of the Li_4_Ti_5_O_12 _powders prepared through the solid-state process. Small and uniformly sized Li_4_Ti_5_O_12 _particles (50 to 100 nm) were obtained even if the calcination temperature was 700°C, which could be attributed to the unique TiO_2 _nanoprecursors with extremely large surface areas. These Li_4_Ti_5_O_12 _powders were further investigated by HRTEM, as shown in Figure 3c. The typical HRTEM image was recorded from a single particle with lattice fringes of approximately 0.496 nm, which corresponded to the (111) interplanar spacing in Li_4_Ti_5_O_12_. The presence of single-phase Li_4_Ti_5_O_12 _was also confirmed from the SAED patterns shown in the inset of Figure 3c.

**Figure 4:**
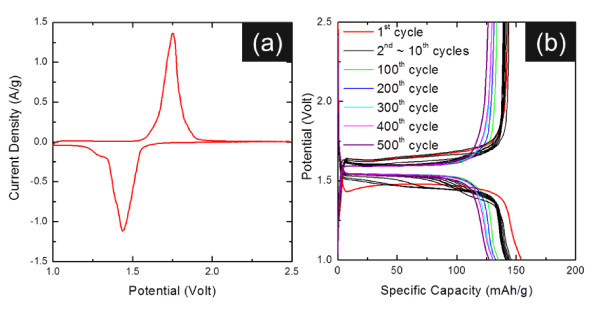
**Electrochemical performance of Li_4_Ti_5_O_12_**. (**a**) A cyclic voltammogram of Li_4_Ti_5_O_12_. (**b**) Charge-discharge profiles of Li_4_Ti_5_O_12 _powders prepared through the solid-state process and subsequently calcined at 700°C for 4 h. (By Jin YH et al.).

Nanostructured electrode materials help in enhancing the performance of Li-ion batteries by providing higher electrode/electrolyte contact areas, shorter Li^+ ^diffusion lengths (*L*) in the intercalation host (smaller time constant (*τ*); *τ *= *L*^2^/2*D*, where *D *is the coupled diffusion coefficient for Li^+ ^and e^-^), and better accommodation of the Li-ion insertion/extraction strain [8,9]. Figure 4 shows the electrochemical activity of nano-Li_4_Ti_5_O_12 _powders that were prepared through the solid-state process. These CV measurements were carried out during the first cycle using a half cell with Li metal foil as the negative electrode, operating at 0.3 mV/s. Clear cathodic and anodic peaks appeared at approximately 1.46 and 1.7 V, respectively, for the Li intercalation/deintercalation, in accordance with the pair of peaks reported for Li_4_Ti_5_O_12 _powders [10]. The following electrochemical reaction of Li_4_Ti_5_O_12 _with Li has been suggested [11]:

$$\text{L}{\text{i}}_{\text{4}}\text{T}{\text{i}}_{\text{5}}{\text{O}}_{\text{12}}+\text{ 3 L}{\text{i}}^{+}+\text{ 3 }{\text{e}}^{-}↔\text{ L}{\text{i}}_{\text{7}}\text{T}{\text{i}}_{\text{5}}{\text{O}}_{\text{12}}.$$

Figure 4b shows the galvanostatic cycling characteristics of nano-Li_4_Ti_5_O_12 _powders that were prepared through the solid-state process. The first discharge capacity was 154 mAh g^-1 ^over a voltage window of 1.0 to 2.5 V at a current rate of 1 C (175 mAh g^-1^; here, C is defined as three Li ions per hour and per formula unit of Li_4_Ti_5_O_12 _on the basis of the above equation). The reversible capacities were observed to be 135, 133, 131, 130, and 128 mAh g^-1 ^after 100, 200, 300, 400, and 500 cycles, respectively. Indeed, it is interesting to note that the nano-Li_4_Ti_5_O_12 _electrode in this study shows superior long-term cyclability and negligible variation in reversible capacity upon cycling (0.013% fading per cycle between 100 and 500 cycles).

**Figure 5:**
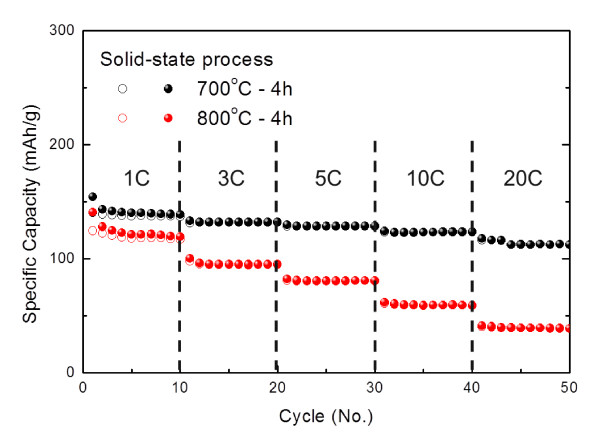
**Rate capability of Li_4_Ti_5_O_12_**. Cycling behavior at different C values for Li_4_Ti_5_O_12 _powders prepared through the solid-state process and subsequently calcined at 700°C and 800°C for 4 h. Solid and open circles indicate discharge and charge capacities, respectively. (By Jin YH et al.).

Figure 5 shows the rate capability of the nano-Li_4_Ti_5_O_12 _powders that were prepared through the solid-state process, for up to 20 C. The cells were charged and discharged at 1 C for the first 10 cycles, and then, the rate was increased in stages to 20 C. At a rate of 20 C, the capacity of the nano-Li_4_Ti_5_O_12 _powders was still high: 112 mAh g^-1^. This outstanding performance at high rates was much better than that afforded by any of the various types of Li_4_Ti_5_O_12 _nanostructures such as nanowires and nanoparticles [3,12,13]. In particular, the nano-Li_4_Ti_5_O_12 _powders calcined at 700°C exhibited better long-term cyclability as well as superior rate capabilities than those calcined at 800°C (Figure 5), possibly a result of the nanosize effect of the small particle size and large surface area.

## Conclusion

In summary, spinel-type nano-Li_4_Ti_5_O_12 _particles were synthesized by a solid-state process from a large-surface-area TiO_2 _precursor and subsequent calcination at 700°C. The average particle size of these nano-Li_4_Ti_5_O_12 _particles was 50 to 100 nm. High Li electroactivity was confirmed by CV experiments. The nano-Li_4_Ti_5_O_12 _particles calcined at 700°C showed a high Li storage capacity of 128 mAh g^-1 ^after 500 cycles at 1 C and superior cycle performance (112 mAh g^-1^) even at a high rate of 20 C. The enhanced reversible capacity and cycling performance were attributed to the formation of highly crystalline, uniform nanoparticles, which make this nano-Li_4_Ti_5_O_12 _a potential host material for high-powder Li-ion batteries.

## Competing interests

The authors declare that they have no competing interests.

## Authors' contributions

Y-HJ carried out the TiO_2 _and Li_4_Ti_5_O_12 _sample preparation and drafted the manuscript. K-MM and H-WS fulfilled the electrochemical analyses. S-DS, I-SH, and K-SP participated in the microstructural analysis. D-WK designed the study, led the discussion of the results, and participated in writing the manuscript. All authors read and approved the final manuscript.
